# Gene Therapy in Cardiovascular Research

**Published:** 2012-10-07

**Authors:** Arpad Tosaki

**Affiliations:** 1 Department of Pharmacology, Faculty of Pharmacy, Health and Science Center, University of Debrecen, Debrecen, Hungary

**Keywords:** Cardiovascular Research, Molecular Biology, Gene Therapy

In less than 40 years of the first human gene being isolated, which involves putting isolated and manipulated DNA (genes) back into individuals for treatments of genetic diseases. Gene therapy is now more widely accepted to encompass all forms of therapeutic intervention in which DNA is used to alter the overall pattern of gene expression within an organ. Potential applications of gene therapy are widespread and implications for the management of various human diseases are becoming available. Approaches are being developed to overcome difficulties and identify a number of potential applications (1). The management of cardiovascular diseases includes the treatment of genetic diseases related to disorders of lipid metabolism and of acquired disease focusing on atherosclerosis and restenosis following angioplasty, thus, the introduction of DNA into the arterial wall.

It is now relatively well accepted that much of human disease can and should be treated at the level of underlying genetic manipulation. Much of the theoretical basis of gene therapy are going to be worked out step by step (2). What we face now is the difficult task of developing the technology required to put these ideas into an actual clinical situation. Although it is relatively easy to get DNA into cells under experimental conditions, it remains much more difficult to do so with the efficiency required for therapy in clinics. What then are the implications for cardiovascular disease?

Several approaches have also been made to modify cell phenotype including inhibition of gene function associated with cell proliferation and to arrest the progress of atherosclerosis and restenosis, and the local production of growth factors to promote angiogenesis (e.g., VEGF. These procedures are of potential use in the treatment of peripheral vascular diseases and myocardial ischemia. Although DNA is taken up by cardiac myocytes relatively efficiently, it is currently difficult to envisage global treatment of different parts of a diseased myocardium. For instance, the direct injection of genes leads to highly localized DNA uptake; adenovirus provides potential for more global DNA transfer but elicits a strong immune response in the body.

There is an increasing interest in the molecular regulation of introduced gene(s) to elicit regionally specific effects i.e., directed for to regions of hypoxia or other stress. Approaches most likely to succeed in gene therapy remain a subject for speculation and based on experimental findings. We should also take caution and not only overestimate the importance of gene therapy at this early, but promising stage. These are exciting times but it takes additional and substantial effort before gene therapy makes real impact in humans. What appears certain is that cardiovascular diseases will be a major of therapeutic applications.
